# Aging‐related cell type‐specific pathophysiologic immune responses that exacerbate disease severity in aged COVID‐19 patients

**DOI:** 10.1111/acel.13544

**Published:** 2022-01-12

**Authors:** Yuan Hou, Yadi Zhou, Lara Jehi, Yuan Luo, Michaela U. Gack, Timothy A. Chan, Haiyuan Yu, Charis Eng, Andrew A. Pieper, Feixiong Cheng

**Affiliations:** ^1^ Genomic Medicine Institute Lerner Research Institute Cleveland Clinic Cleveland Ohio USA; ^2^ Quantitative Health Sciences, Lerner Research Institute Cleveland Clinic Cleveland Ohio USA; ^3^ Neurological Institute Cleveland Clinic Cleveland Ohio USA; ^4^ Division of Health and Biomedical Informatics Department of Preventive Medicine Clinical and Translational Sciences Institute and Center for Health Information Partnerships Northwestern University Evanston Illinois USA; ^5^ Florida Research and Innovation Center Cleveland Clinic Port Saint Lucie Florida USA; ^6^ Center for Immunotherapy and Precision Immuno‐Oncology Cleveland Clinic Cleveland Ohio USA; ^7^ Weill Institute for Cell and Molecular Biology Cornell University Ithaca New York USA; ^8^ Department of Computational Biology Cornell University Ithaca New York USA; ^9^ Department of Molecular Medicine, Cleveland Clinic Lerner College of Medicine Case Western Reserve University Cleveland Ohio USA; ^10^ Department of Genetics and Genome Sciences Case Western Reserve University School of Medicine Cleveland Ohio USA; ^11^ Case Comprehensive Cancer Center Case Western Reserve University School of Medicine Cleveland Ohio USA; ^12^ Harrington Discovery Institute University Hospitals Cleveland Medical Center Cleveland Ohio USA; ^13^ Department of Psychiatry Case Western Reserve University Cleveland Ohio USA; ^14^ Geriatric Psychiatry GRECC Louis Stokes Cleveland VA Medical Center Cleveland Ohio USA; ^15^ Institute for Transformative Molecular Medicine School of Medicine Case Western Reserve University Cleveland Ohio USA; ^16^ Weill Cornell Autism Research Program Weill Cornell Medicine of Cornell University New York New York USA; ^17^ Department of Neuroscience School of Medicine Case Western Reserve University Cleveland Ohio USA

**Keywords:** aging, cellular immunology, COVID‐19, molecular biology of aging, SARS‐CoV‐2

## Abstract

Coronavirus disease 2019 (COVID‐19) is especially severe in aged patients, defined as 65 years or older, for reasons that are currently unknown. To investigate the underlying basis for this vulnerability, we performed multimodal data analyses on immunity, inflammation, and COVID‐19 incidence and severity as a function of age. Our analysis leveraged age‐specific COVID‐19 mortality and laboratory testing from a large COVID‐19 registry, along with epidemiological data of ~3.4 million individuals, large‐scale deep immune cell profiling data, and single‐cell RNA‐sequencing data from aged COVID‐19 patients across diverse populations. We found that decreased lymphocyte count and elevated inflammatory markers (C‐reactive protein, D‐dimer, and neutrophil–lymphocyte ratio) are significantly associated with age‐specific COVID‐19 severities. We identified the reduced abundance of naïve CD8 T cells with decreased expression of antiviral defense genes (i.e., *IFITM3* and *TRIM22*) in aged severe COVID‐19 patients. Older individuals with severe COVID‐19 displayed type I and II interferon deficiencies, which is correlated with SARS‐CoV‐2 viral load. Elevated expression of SARS‐CoV‐2 entry factors and reduced expression of antiviral defense genes (*LY6E* and *IFNAR1*) in the secretory cells are associated with critical COVID‐19 in aged individuals. Mechanistically, we identified strong TGF‐beta‐mediated immune–epithelial cell interactions (i.e., secretory‐non‐resident macrophages) in aged individuals with critical COVID‐19. Taken together, our findings point to immuno‐inflammatory factors that could be targeted therapeutically to reduce morbidity and mortality in aged COVID‐19 patients.

## Graphical abstract

Our study identified that elevated inflammatory markers (C‐reactive protein, D‐dimer, and neutrophil–lymphocyte ratio) are significantly associated with age‐specific COVID‐19 severities. Mechanistically, we identified that reduced abundance of naïve CD8 T cells and type I and II interferon deficiencies are associated with severity of COVID‐19 in aged individuals. We identified strong TGF‐beta‐mediated immune–epithelial cell interactions in aged individuals with critical COVID‐19.

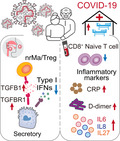

## 1 INTRODUCTION

Coronavirus disease 2019 (COVID‐19), a global pandemic caused by severe acute respiratory syndrome coronavirus 2 (SARS‐CoV‐2), has been diagnosed in more than 284 million people globally, with 5.4 million deaths since December 2019 (data on December 30, 2021). Although a serious risk at any age, SARS‐CoV‐2 infection is particularly debilitating and deadly for aged patients, defined in this study as 65 years and older (Channappanavar & Perlman, 2020; Clay et al., 2014; Davies et al., 2020; O'Driscoll et al., 2021). The molecular basis of this aging‐related vulnerability is an important area of investigation as it is currently poorly understood.

Impaired and dysregulated host immunities, including both innate and adaptive immunities, have been hypothesized as age‐based factors in COVID‐19 disease severity (Brodin, 2021; Channappanavar & Perlman, 2020). Compared to younger individuals with COVID‐19, aged individuals show disrupted antigen‐specific adaptive immunity to SARS‐CoV‐2, such as reduced coordination of CD4‐CD8 T‐cell responses (Rydyznski Moderbacher et al., 2020). In addition, aged individuals typically produce a less robust type I interferon (IFN) response to flu virus infections (Molony et al., 2017), indicating compromised cellular antiviral defense in innate immunity. Indeed, 13% of aged patients with life‐threatening COVID‐19 display inborn errors in autoantibodies against type I IFN immunity (Bastard et al., 2020). In addition, aberrant immunosenescence and inflammation also play crucial roles in age‐medicated COVID‐19 morbidity and mortality (Domingues et al., 2020). For example, senescent cells become hyper‐inflammatory in response to pathogen‐associated molecular patterns, and senolytics reduce COVID‐19 mortality in aged mice (Camell et al., 2021). Based on these findings, we sought to systematically identify whether there are specific immuno‐inflammatory determinants that promote age‐associated COVID‐19 severity.

## 2 RESULTS

### 2.1 Severe outcomes in aged COVID‐19 patients

**FIGURE 1:**
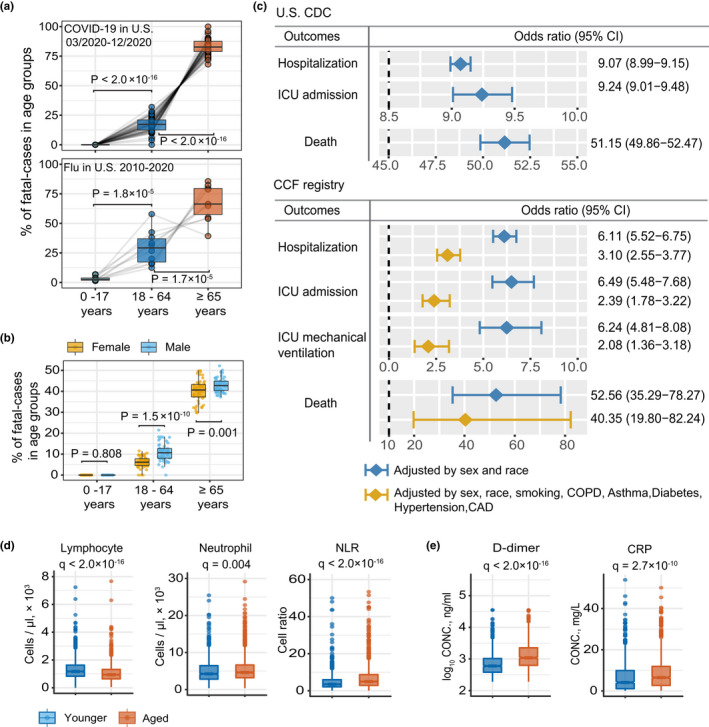
Epidemiological data analysis between aged and younger COVID‐19 patients. (a) The percentage of fatal cases of COVID‐19 and flu across three age groups. Data source from U.S. CDC. The upper panel shows the percentage of fatal cases of COVID‐19 in the United States. Each dot in the boxplot represents one state. The lower panel shows the percentage of fatal cases of flu from 2010 to 2020. Each dot in the boxplot represents one flu season. Statistical *p*‐value was computed by two‐tailed paired t test. For details about CDC dataset, see Tables S1 and S2. (b) Sex differences in the percentage of fatal cases of COVID‐19 across three age groups. (c) Odds ratio (OR) analysis of U.S. CDC and COVID‐19 registry datasets. U.S. CDC dataset, “Younger” is defined as 20 to 49 years of age (*n* = 2,369,919), and ‘aged’ is defined as >60 years old (*n* = 1,048,011); COVID‐19 registry dataset, “Younger” is defined as 18 to 55 years of age (*n* = 12,651), and ‘aged’ is defined as ≥65 years old (*n* = 32,426). OR >1 indicates aged COVID‐19 patients with increased likelihood of hospitalization, ICU admission, and death. Two colors denote OR models with different adjusted confounders. Features of the COVID‐19 registry dataset are shown in Table S3. (d) and (e) Boxplot show the lab testing values of five inflammatory markers between aged (>65 years, *n* = 1405) and younger (18 to 55 years, *n* = 970) individuals. Adjusted p‐value [*q*] was computed by Mann–Whitney *U* test with Benjamini–Hochberg (BH) multiple testing correction

To begin, we investigated the prevalence of COVID‐19 disease among different age groups with 9 months of data collection. Analysis of U.S. Centers for Disease Control (CDC) epidemiological data from March to December 2020 (Tables S1–S3) revealed that 80.5% of fatal cases occurred in aged patients. Strikingly, this rate was 4.1 times higher than in 18–64 years old (19.5%), and 1653 times higher than in 0–17 years old (0.05%, Figure 1a). Fatality prevalence was influenced by sex in both older and younger groups (Figure 1b). Interestingly, we found that average fatal percentage in aged COVID‐19 patients is 16% higher than that of influenza (Flu) (Table S2), indicating that COVID‐19 is more hazard for aged individuals than Flu.

Next, we used odds ratio (OR) adjusted for confounding factors to further evaluate the association between aging and four types of COVID‐19 outcomes: hospitalization, intensive care unit (ICU) admission, ICU mechanical ventilation, and death. Specifically, we analyzed sex‐ and race‐adjusted OR values in 3,417,930 COVID‐19‐positive cases (*n* = 2,369,919 in young individuals, 20–49 years old) and *n* = 1,048,011 in aged individuals (>60 years old) (see Method; Table S3) from the U.S. CDC database. Here, aged individuals showed significantly increased likelihood of COVID‐19‐related hospitalization (OR = 9.07, 95% confidence interval [CI] 9.99–9.15; Figure 1c), ICU admission (OR = 9.24, 95% CI 9.01–9.48), and death (51.15, 95% CI 49.86–52.47; Figure 1c).

To further account for disease comorbidities, we next computed OR across different age groups using a large COVID‐19 registry database with 12,651 aged (≥65 years) and 32,426 younger individuals (20–55 years old) (Figure 1c, Table S4, see Methods). Specifically, we tested the OR Model‐2, which is adjusted for sex, race, smoking, and five common disease comorbidities (Guan et al., 2020; Yang, Zheng, et al., 2020) (hypertension, diabetes, coronary artery disease [CAD], asthma, chronic obstructive pulmonary disease [COPD], and emphysema). Here, we again found that aged individuals had significantly greater likelihood of COVID‐19‐related hospitalization (OR = 3.10, 95% CI 2.55–3.77), ICU admission (OR = 2.39, 95% CI 1.78–3.22) (Figure 1c), and death (OR = 40.35, 95% CI 19.80–82.24). Subsequent Kaplan–Meier analysis further revealed an elevated cumulative hazard for hospitalization (*p* < 0.0001, log‐rank test; Figure S1a), including longer duration of hospitalization (average duration = 8.9 days; *p* = 1.4 × 10^−15^, Mann–Whitney *U* test; Figure S1b), in COVID‐19 patients. Taken together, our findings confirm an elevated likelihood of severe outcomes in aged COVID‐19 patients has compared with younger patients, even when adjusted for all possible confounding factors.

### 2.2 Elevated inflammatory responses in aged COVID‐19 patients

As severe COVID‐19 patients have been reported to have lower lymphocyte count (Yang, Liu, et al., 2020) and higher C‐reactive protein (CRP) (Manson et al., 2020), we examined the Cleveland Clinic COVID‐19 registry for differences in inflammatory biomarkers as a function of aging. Here, we found lower peripheral lymphocytes (adjusted *p*‐value [*q*] <2.0 × 10^−16^, Mann–Whitney *U* test with Benjamini–Hochberg multiple test correction; Figure 1d) and higher circulating neutrophils in hospitalized aged COVID‐19 patients (*q* = 0.004; Figure 1d), compared with younger patients. We also found that the neutrophil–lymphocyte ratio (NLR), a marker of systemic inflammation (Cai et al., 2021), was elevated in aged COVID‐19 patients (*q* < 2.0 × 10^−16^; Figure 1d). In addition, the inflammatory markers D‐dimer (*q* < 2.0 × 10^−16^; Figure 1e) and C‐reactive peptide (CRP) (*q* = 2.7 × 10^−10^; Figure 1e) were also significantly increased in hospitalized aged patients compared with hospitalized young COVID‐19 patients. Those findings motivate us to inspect heterogeneities of immune cells using large‐scale immune cell phenotypic profiles and single‐cell transcriptomics datasets under a multimodal genomic analytic framework.

### 2.3 Elevated pro‐inflammatory cytokine expression in aged COVID‐19 patients

**FIGURE 2:**
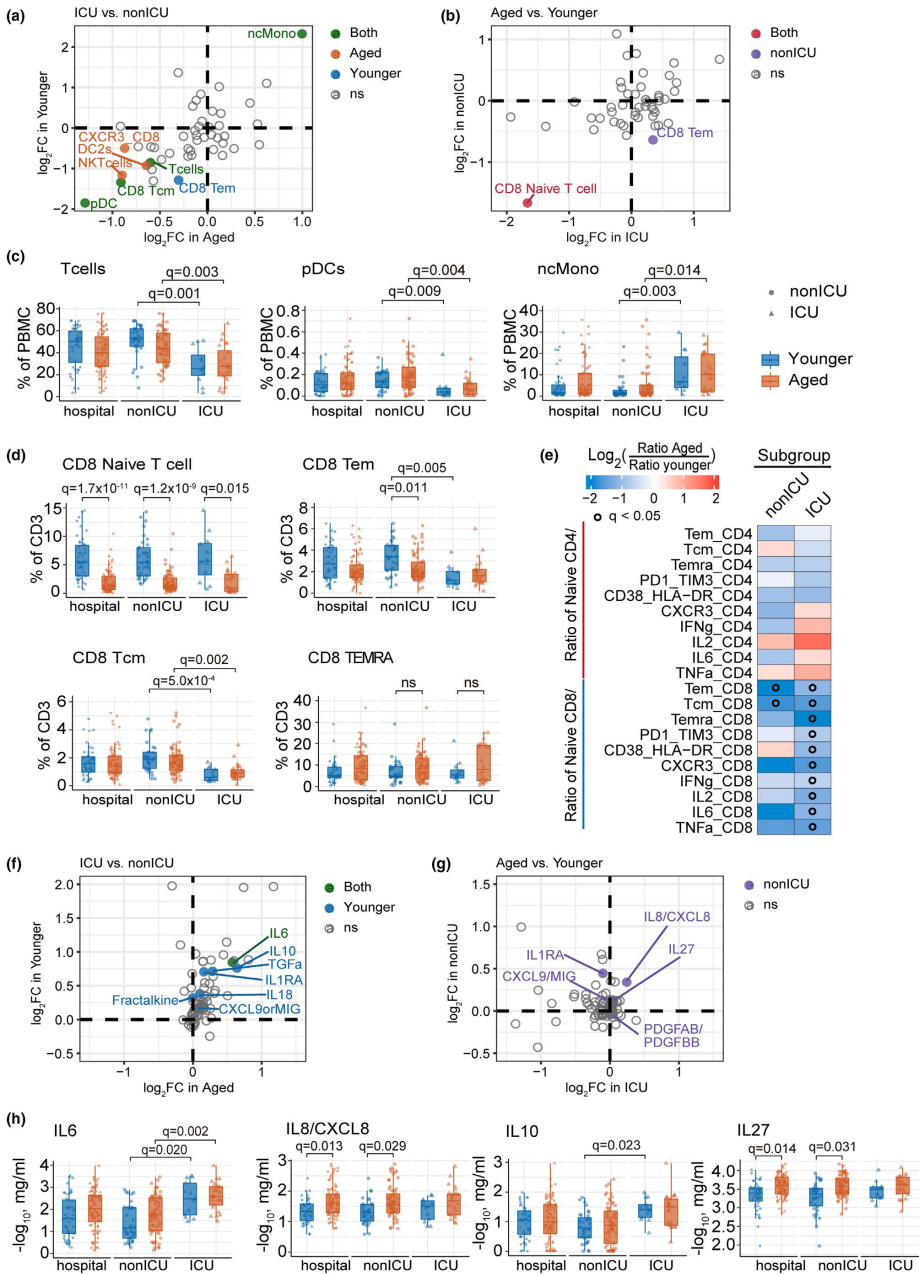
Deep immune‐profiling of aged and younger patients with COVID‐19. (a) and (f) Scatterplots show the differential immune cell type (a) and cytokines (**f**) between ICU (*n* = 39 samples, aged *n* = 26, younger *n* = 13) versus non‐ICU (105 samples, aged *n* = 68, younger *n* = 37) COVID‐19 patients. The cell flow and cytokine profiling datasets were collected from a recent study (Takahashi et.al, 2020) (see Method). Y‐axis and X‐axis show the log2(Fold Change [FC]) in younger and aged subpopulations. The pairwise comparison group is ICU vs. non‐ICU patients with COVID‐19. Solid green dots denote significantly different cell types or cytokines in both younger and aged patients. Solid blue and orange dots denote significantly different cell types or cytokines in younger and aged patients, respectively. (b) and (g) Scatterplots show the differential immune cell type (b) and cytokines (g) in aged (*n* = 94 samples) versus younger (50 samples) COVID‐19 patients. Y‐axis and X‐axis show the log2FC in ICU and non‐ICU subpopulations. The pairwise comparison group is aged vs. younger patients with COVID‐19. Solid red dots denote significantly different cell types or cytokines in both ICU and non‐ICU patients. Solid purple dots denote significantly different cell types or cytokines in non‐ICU patients. (c) The abundance of major immune cell types in PBMC and (d) subtypes of CD8+ T cells in all CD3‐positive cells. Statistical adjusted *p*‐value (*q*) was computed by Mann–Whitney *U* test with BH multiple testing correction (e) Heatmap showing the ratio of naïve vs memory lymphocytes. Gradient color indicated the log2 fold change in average ratio between aged and younger in non‐ICU or ICU subgroup, respectively. Black circle indicates *q* < 0.05. (h) The abundance of four cytokines changes between younger and aged COVID‐19 patients in hospital, ICU, and non‐ICU groups

We next examined peripheral immune cell profiles (Takahashi et al., 2020) of hospitalized aged and younger COVID‐19 patients by querying a publicly available dataset of 12 major immune cell types (% peripheral blood mononuclear cells [PBMCs]) and 32 T‐cell subtypes (% CD3, Table S5, see Methods). All markers and cell type/subtype definitions are provided in the original study (Takahashi et al., 2020). There was no difference in abundance of the major immune cell types (e.g., T cells, B cells, natural killer cells, and plasmacytoid dendritic cells [pDC]) between aged and young hospitalized COVID‐19 patients, including those in the ICU (Figure 2a,c and Figure S2a). However, both young and aged COVID‐19 patients with ICU admission had a lower proportion of T cells (younger, *q* = 0.001; older, *q* = 0.003) and pDC (younger, *q* = 0.009; older, *q* = 0.004) (Figure 2a,c), as well as an elevated proportion of non‐classic monocytes (ncMono) (younger, *q* = 0.003; older, *q* = 0.014; Figure 2c), compared with non‐ICU patients. Further analysis of deep phenotyping T‐cell data revealed significantly fewer naïve CD8 T cells in hospitalized aged COVID‐19 patients (*q* = 1.7 × 10^−11^; Figure 2b,d). Naïve CD8 T‐cell‐mediated homeostasis is an important component of antiviral defense (Kaech & Cui, 2012), and the naïve CD8 T‐cell receptor repertoire is negatively correlated with age in COVID‐19 patients (Ren et al., 2021). Thus, reduced abundance of naïve CD8 T cells may be associated with COVID‐19 severities in aged individuals.

We next turned to investigate the ratio of naïve vs. other T‐cell subsets and natural killer T (NKT) vs. natural killer (NK) cells (Figure 2e). We found that the ratio of CD8 naïve T cell with multiple CD8 T‐cell subsets was significantly decreased in aged ICU individuals compared with younger patients (Figure 2e). The ratio of CD8 naïve T cell with memory CD8 T cell (Tem and Tcm) was significantly reduced in aged COVID‐19 patients in both ICU and non‐ICU. In particular, the ratios of CD8 naïve T cell with PD1‐TIM3‐CD8 T cell and CD38‐HLA‐DR CD8 T cell were significantly decreased in aged COVID‐19 patients compared with younger patients in ICU, not in non‐ICU. The gene PD1 and TIM3 are makers for CD8 T‐cell exhaustion, and an elevated PD1 in exhausting T cells was highly associated with severe COVID‐19 (Neidleman et al., 2021). CD38 and HLA‐DR are markers for CD8 T‐cell activation, and an accumulated activation of HLA‐DR is associated with severe COVID‐19 (Neidleman et al., 2021; Quinn et al., 2018). Altogether, reduced ratio of naïve CD8 T cells and CD8 memory T cell in severe COVID‐19 (Figure 2e) could be explained by non‐specific memory T‐cell activation and dysfunctional immune responses (de Candia et al., 2021) in aged individuals. Yet, the ratio of CD4 naïve T cells with other CD4 T sub‐cell type and NKT with NK has no significant difference between aged and younger patients in both ICU and non‐ICU.

Next, we compared the plasma profile of 71 cytokines and chemokines (Takahashi et al., 2020) between hospitalized aged and younger COVID‐19 patients (Table S5). Historically, increased IL‐6, IL‐8, IL‐10, and IL‐27 levels have been associated with severe COVID‐19 (Del Valle et al., 2020; Lu et al., 2021). Here, we found that elevated expression of IL‐8 (also named CXCL8) and IL‐27 in aged COVID‐19 patients (*q* = 0.013; Figure 2h). As IL‐8 is a pro‐inflammatory cytokine via recruiting and activating neutrophils (Bickel, 1993), its elevation is consistent with our previously noted elevated neutrophil count and NLR in hospitalized aged COVID‐19 patients (Figure 1d). Furthermore, younger, but not aged, COVID‐19 ICU patients also showed elevated IL‐10 (Figure 2h), a key feature of cytokine storm (Huang et al., 2020; Zhao et al., 2020). In addition, elevated IL‐6 was observed in both younger (*q* = 0.020) and aged ICU patients, (*q* = 0.002), compared with non‐ICU patients (Figure 2h). Altogether, severe COVID‐19 patients show distinct age‐related cytokine profiles: (a) Aged COVID‐19 patients in hospitalization have elevated level of IL‐6, IL‐8, and IL‐27, while (b) younger patients with ICU have elevated IL‐6 and IL‐10 expression. These results indicate that heterogeneous inflammatory cytokine expression between aged and younger COVID‐19 patients may mediate age‐related hospitalization and ICU admission.

### 2.4 Reduced naïve CD8 T cells in aged severe COVID‐19 patients

**FIGURE 3:**
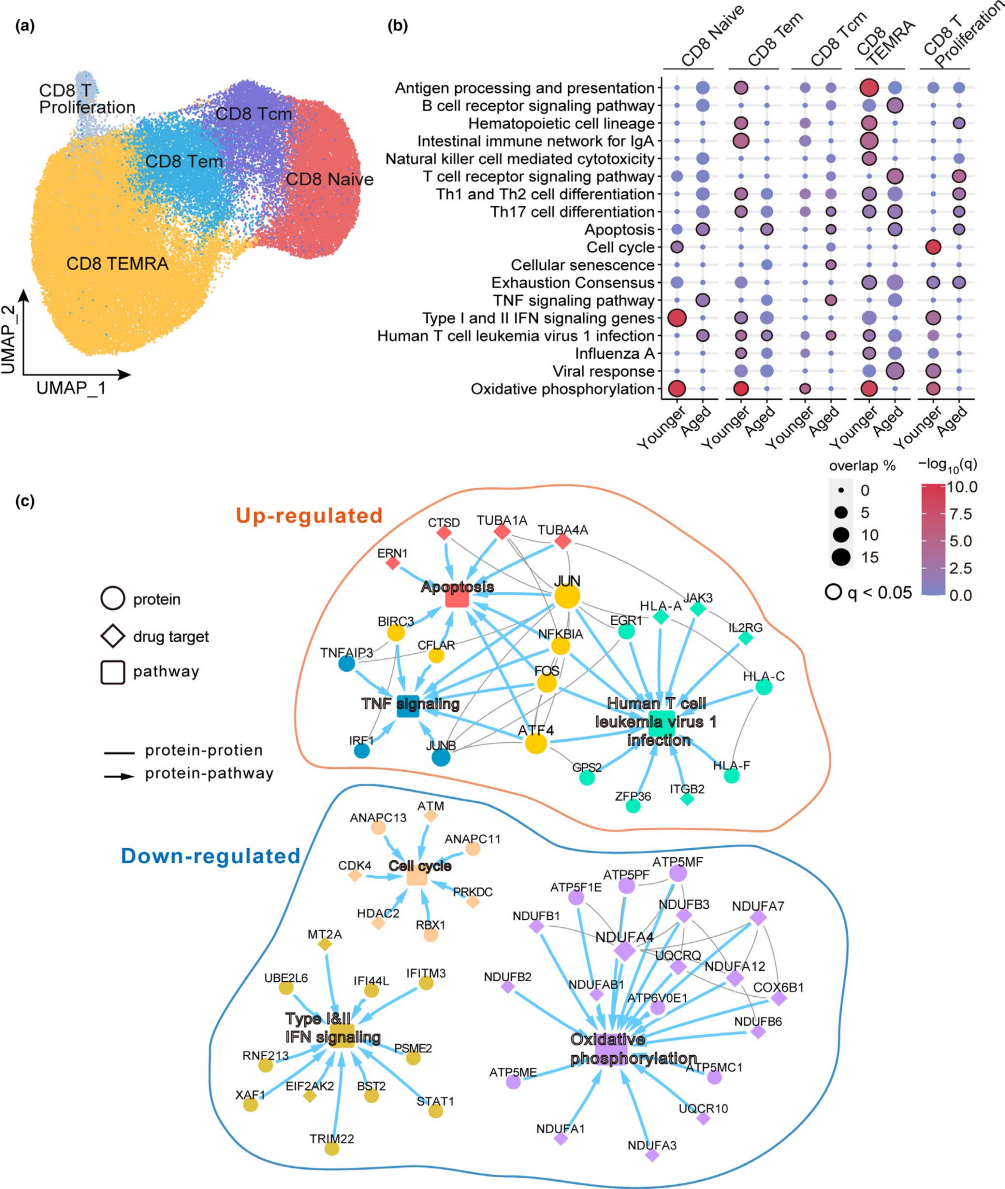
Single‐cell transcriptome of CD8 T cells in aged COVID‐19 patients. (a) UMAP plot displays five identified CD8 T‐cell subpopulations. The single‐cell transcriptomic dataset (25 of Severe\Critical COVID‐19 patients, aged *n* = 12, younger *n* = 13) was collected from a recent study (Stephenson et al., 2021) (Table S1 and Method). (b) Pathway enrichment analysis across five CD8 T‐cell subtypes. Black circle indicates *q* < 0.05. (c) A highlighted protein–protein interaction subnetwork for age‐biased differentially expressed genes in CD8 naïve T cells from patients with critical COVID‐19. The colors for nodes and edges represent different immune pathways

Because we observed loss of CD8 naïve T cells and T effector memory cells in hospitalized aged COVID‐19 patients (Figure 2 b, d), we examined a publicly available single‐cell transcriptomic dataset of CD8 T cells from 25 severe/critical COVID‐19 patients (aged *n* = 12; younger *n* = 13) (Stephenson et al., 2021) in order to search for aging‐related molecular mechanisms in a cell type‐specific manner. Uniform Manifold Approximation and Projection (Becht et al., 2019) (UMAP) analysis revealed five distinct CD8 sub‐clusters (Figure 3a and Figure S3) based on biomarkers provided from the original literature (See Method, Stephenson et al., 2021), including naïve CD8, T central memory (Tcm), Tem, CD8 proliferation, and CD8 terminal effector T cell (also designated as TEMRA, Thome et al., 2014). We found that aged and younger patients with severe COVID‐19 showed age‐dependent immune pathway profiles across five CD8 subtypes. For example, type I and II IFN signaling showed decreased effect in CD8 naïve T cells, CD8 Tem, and CD8 proliferation T cells isolated from PBMC in aged severe COVD‐19 patients, not in younger patients (Figure 3b). In addition, the antigen processing and presentation pathway showed decreased effect in CD8 Tem and CD8 TEMRA in aged patients as well. Our finding indicates that type I and II IFN signaling and antigen processing and presentation pathways are age‐related immune pathways associated with COVID‐19 disease severity. Yet, Th17 cell differentiation pathway of CD8 TEMRA and exhaustion consensus of CD8 T proliferation cells were activated in both aged and younger patients with severe COVID‐19.

We next turned to investigate the molecular network in CD8 naïve T cells. Comparing to severe young COVID‐19 patients, up‐regulated genes (*q* < 0.05, log‐fold change >0.1) in CD8 naïve T cells from aged patients formed a network module (the largest connected component) in the human protein–protein interactome (Figure 3c). This age‐specific network module was significantly enriched in several pathways, including apoptosis (*q* = 0.013), human T‐cell leukemia virus 1 infection (*q* = 0.013), and TNF signaling (*q* = 0.014; Figure 3c). In particular, the apoptosis gene cathepsin D (Cocchiaro et al., 2016) (*CTSD*) was highly expressed in naïve CD8 T cells from aged severe COVID‐19 patients (*q* < 2.0 × 10^−16^). Down‐regulated genes, such as interferon‐stimulated genes *IFITM3* and *TRIM22*, in CD8 naïve T cells from aged COVID‐19 patients were enriched in type I and II IFN signaling pathways (Figure 3c). In addition, the transcription factor *STAT1*, an important downstream factor in type I and II IFN signaling pathways (Hu & Ivashkiv, 2009), was down‐regulated in CD8 naïve T cells in aged COVID‐19 patients (Figure 3c). Notably, the SARS‐CoV‐1 NSP1 protein impedes type I and II IFN signaling (Matsuyama et al., 2020) by attenuating STAT1 phosphorylation (Wathelet et al., 2007). Thus, IFN deficiencies in CD8 naïve T cells may contribute to increased severity of COVID‐19 disease in aged patients.

### 2.5 Interferon deficiencies correlate with SARS‐CoV‐2 viral load in aged patients

**FIGURE 4:**
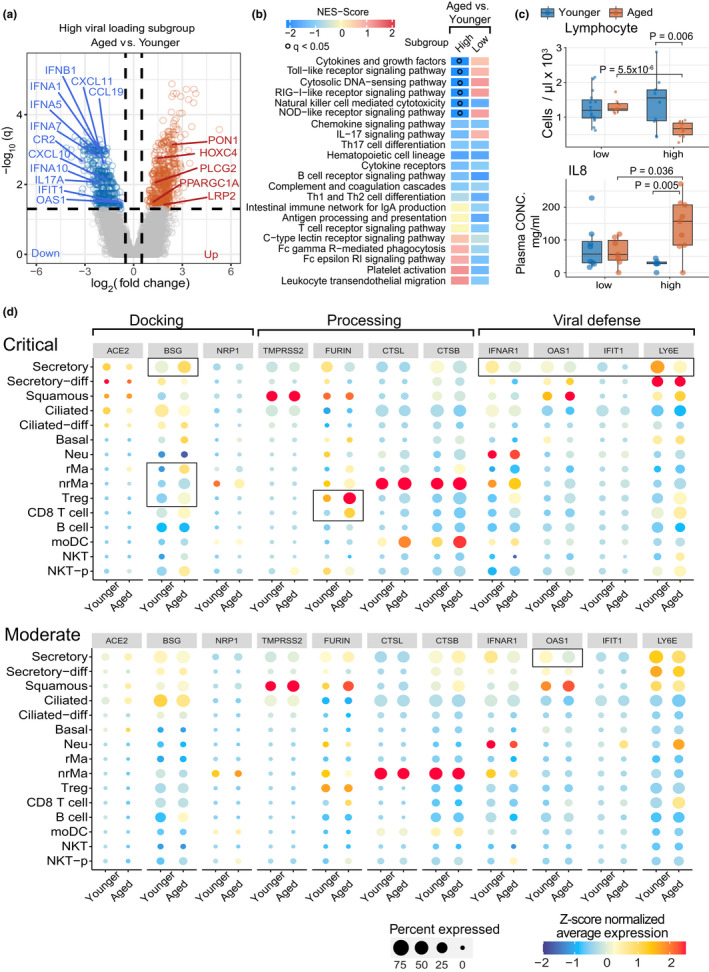
Analysis of SARS‐CoV‐2 viral load and related entry gene expression in nasal tissues. (a) Volcano plot showing the differential genes of bulk RNA‐sequencing data in aged versus younger patients in high viral load nasal tissues. A publicly available bulk RNA‐seq dataset of 147 nasal samples (Lieberman et al., 2020) was used, including 61 aged patients (high [*n* = 27] and low [*n* = 34] viral load) and 86 younger patients (high [*n* = 46] and low [*n* = 40] viral load). (b) Gene‐set enrichment analysis (GSEA) of 22 immune pathways for differential genes of aged vs. younger in high or low viral load subgroups. The gradient color bar shows the NES score (see Method). NES score >0 and *q* < 0.05 indicate that up‐regulated differential expressed genes (DEGs) in aged vs. young are significantly enriched in immune pathways, while NES score <0 and *q* < 0.05 indicate down‐regulated DEGs in aged vs. young are significantly enriched in immune pathways. Black dots denote *q* < 0.05. (c) Boxplot showing the lab testing data changes in aged and younger COVID‐19 patients with high (>4.5 log10 RNA copies/ml) and low (<4.5 log10 RNA copies/ml) viral load(Pekosz et al., 2021; Yang, Jiang, et al., 2020). (d) SARS‐CoV‐2‐related entry gene expression profile across 15 cell types of nasal tissue between aged and younger patients. The size of dot denotes the percentage of the positive cell which expressed the tested genes. The gradient color bar represents the *z*‐score scaled average expression of genes in each cell type

To further investigate the relationship between viral load and COVID‐19 disease severity, we analyzed bulk RNA‐seq data from nasopharyngeal samples (Lieberman et al., 2020) (see Methods). Consistent with our findings in naïve CD8 T cells, expression levels of IFNα genes (*IFNA1*, *IFNA5*, *IFNA7*, and *IFNA8*) were significantly decreased in aged patients with high viral load (Figure 4a). In addition, the expression of *IFNG* was decreased in aged patients with low viral load (Figure S4a). Notably, we found that the IFN‐stimulated antiviral genes (Sadler & Williams, 2008), including *IFIT1* and *OAS1* (2'‐5'‐oligoadenylate synthetase 1), were down‐regulated in aged patients with a higher viral load (Figure 4b). Next, we performed gene set enrichment analysis (GSEA, see Methods) for differentially expressed genes in aged vs. younger individuals with a higher viral load and found downregulation of genes in the innate immune pathways (*q* < 0.05; Figure 4b) of RIG‐I like receptor signaling, Toll‐like receptor signaling, and NOD‐like receptor signaling in aged COVID‐19 patients. RIG‐I‐like receptors senses SARS‐CoV‐2 RNA and subsequently type I IFN production (Onomoto et al., 2010); however, SARS‐CoV‐2 has evolved several mechanisms to blunt IFN induction, including the direct targeting of MDA5 (melanoma differentiation‐associated protein 5), a RIG‐I‐like receptor, by the viral papain‐like protease (PLpro) (Liu et al., 2021). Furthermore, IFN potently inhibits IL‐8 expression (Aman et al., 1993) in viral infection, and we also showed that aged COVID‐19 patients with high viral load exhibit elevated plasma IL‐8 (*p* = 0.005, Mann–Whitney *U* test; Figure 4c). Notably, up‐regulated genes in aged patients with high viral load were not enriched in immune pathways (Figure 4b and Figure S4b), indicating decreased immune ability in response to SARS‐CoV‐2 infection. Taken together, our data show that IFN deficiency is associated with elevated SARS‐CoV‐2 viral load in aged patients.

### 2.6 Age‐dependent increased expression of SARS‐CoV‐2 entry factors

We next investigated age‐ and cell type‐specific expression of SARS‐CoV‐2 entry factors using a single‐cell RNA‐sequencing dataset (Chua et al., 2020) (scRNA‐seq, see Methods) from nasal tissue of critical (*n* = 11) and moderate COVID‐19 patients (*n* = 8, see Methods). In total, the scRNA‐seq dataset contained 115,895 cells across 15 well‐annotated cell types within two main cell populations: epithelial cells (six cell types) and immune cells (nine cell types).

We found that secretory and ciliated cells in aged COVID‐19 patients display reduced abundance of angiotensin‐converting enzyme‐2 (ACE2), a key SARS‐CoV‐2 docking receptor (Yan et al., 2020) (Figure 4d). However, the more recently identified SARS‐CoV‐2 docking receptor basigin (Wang et al., 2020) (BSG or CD147) was expressed in 95% of secretory cells in aged patients with critical COVID‐19 (Figure 4d and Table S6); furthermore, BSG and CD147 showed elevated expression in Treg (regulatory T cell) and CD8 T cells (Figure 4d) as well. We also found that the S protein priming proteases TMPRSS2 (Hoffmann et al., 2020) and FURIN (Zhao et al., 2020) were highly expressed in epithelial cells in critical and moderate COVID‐19, with no differences between aged and young patients (Figure 4d and Table S6). However, FURIN levels were increased in several immune cell types, including Treg and CD8 T cells, in aged patients with critical COVID‐19 (Figure 4d). Taken together, our results suggest that elevated expression of two SARS‐CoV‐2 factors (BSG and FURIN) in Treg and CD8 T cells may contribute to the increased susceptibility of aged patients to COVID‐19.

### 2.7 Increased immune–epithelial cell interactions in aged COVID‐19 patients

**FIGURE 5:**
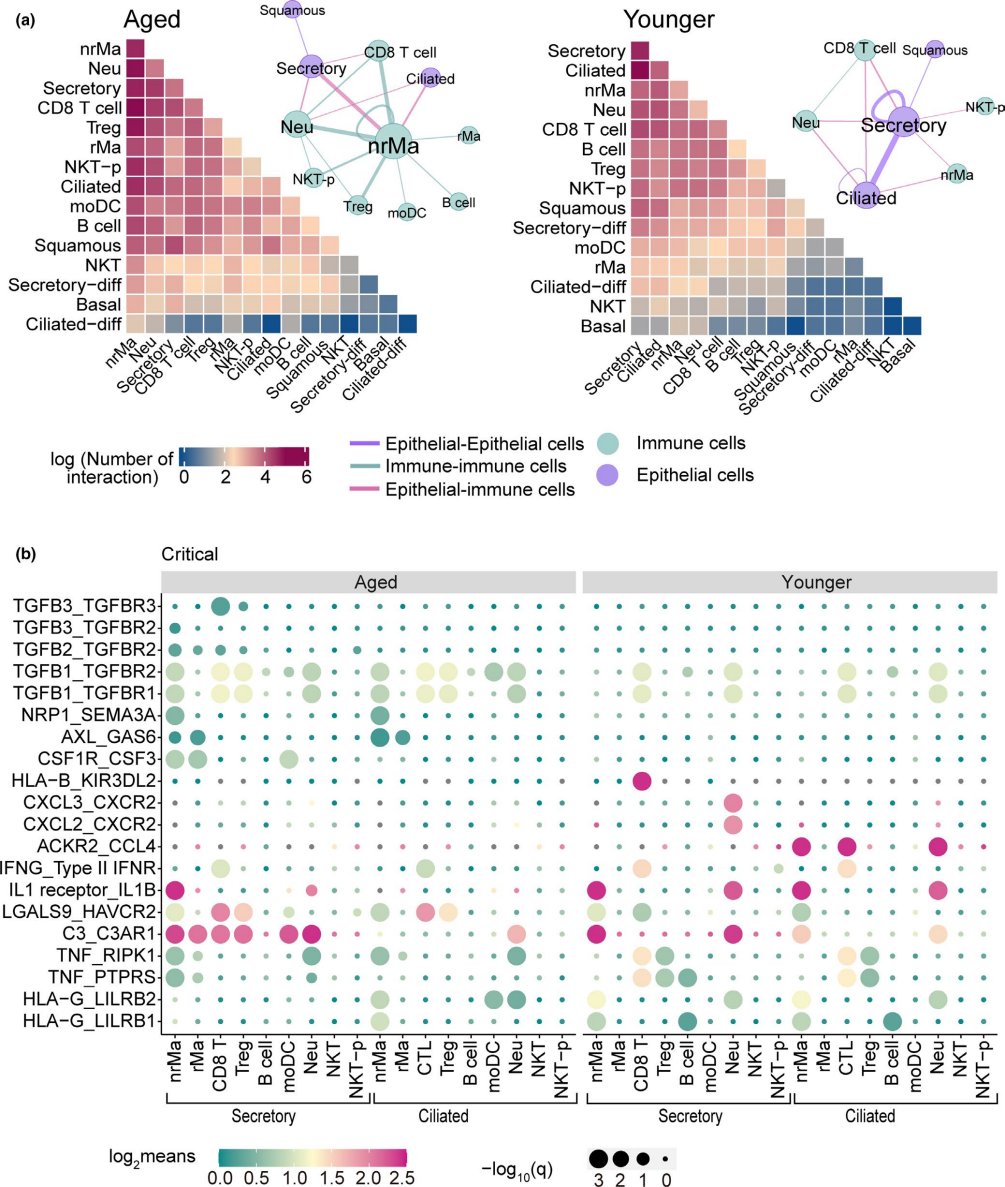
Distinct epithelial‐immune cell interaction profile in aged and younger patients with critical COVID‐19. (a) Heatmap showing the total log‐scaled interaction number between epithelial–immune cells in critical COVID‐19 disease. Aged group, *n* = 3 patients, younger group, *n* = 5 patients. The cell–cell interaction network depicted all significant cell pairs in which the number of ligand–receptor interaction >50 (permutation test with BH multiple testing correction, *q* < 0.05). Edge size denotes the number of significant ligand–receptor interactions between two cell types. Different colors indicate the immune or epithelial cell types. (b) Dot plot showing significant ligand–receptor interactions between epithelial–immune cell interaction in critical COVID‐19 disease. The circle size indicates ‐log_10_(*q*), and gradient color bar shows the log2‐scaled means of average expression of interacted cell pair

To further investigate the immunological mechanisms underlying age‐associated COVID‐19 outcomes, we performed Gene‐set enrichment analysis (GSEA) to explore transcriptomic signatures on 22 immune pathways across 15 cell types derived from nasal tissue (see Methods). Here, we observed distinct immune responses between older and younger individuals with critical or moderate COVID‐19 (Figure S6) in epithelial and immune cell types. We further used CellphoneDB (Efremova et al., 2020) to quantify ligand–receptor interactions between epithelial and immune cells and found an elevated number of significant ligand–receptor interactions involved in immune–epithelial interactions (*q* < 0.05, permutation test with BH multiple testing correction (Benjamini & Hochberg, 1995), Table S7) in aged patients with critical COVID‐19 (Figure 5a). In addition, we also found a stronger immune–epithelial cell interaction network in aged patients. In particular, we noted that secretory‐non‐resident macrophages (nrMa) displayed the highest connection with other cell types in aged patients with critical COVID‐19 (Figure 5a).

We next analyzed ligand–receptor interactions of secretory/ciliated–immune cells in aged and younger patients with critical COVID‐19 (Figure 5b). We found elevated expression of TGF‐β genes (*TGFB1*, *TGFB2*, and *TGFB3*) and their interacting partners (i.e., *TGFBR2* and *TGFBR3*, *q* < 0.05; Figure S7 and Table S7) in nrMa cells and Treg. Of note, TGF‐β has previously been shown to regulate the chronic immune response to SARS‐CoV‐2 in severe COVID‐19 patients (Ferreira‐Gomes et al., 2021). Thus, TGF‐β‐mediated strong secretory and nrMa cell interaction may explain the longer duration of hospitalization in aged COVID‐19 patients (Figure S1b).

We also observed distinct immune–epithelial cell interactions in younger COVID‐19 patients. For example, secretory and CD8 T cells expressed high levels of several ligand–receptor pairs, including HLA‐B–KIR3DL2, TNF–RIPK1, and TNF–PTPRS (*q* < 0.05, permutation test), and secretory and Neu cells highly co‐expressed CXCL2/3 and CXCR2 (*q* < 0.05, permutation test). In addition, we found that secretory/ciliated–CD8 T cells showed a similar IFNG–IFNGR pattern, while the expression level in aged patients was much lower (Figure 5b). In particular, secretory/ciliated–CD8 T‐cell interaction in younger patients showed strong IFNG–IFNGR interaction compared to aged patients with moderate COVID‐19 (Figure S6). In summary, these observations revealed that immune–epithelial cell interactions are associated with critical COVID‐19 in aged patients. In particular, reduced expression of IFNR signaling is associated with greater severity of COVID‐19 in aged individuals (Figure 4a).

## 3 DISCUSSION

This study provides a comprehensive analysis of immune profiles in aged and younger COVID‐19 patients using large, electronic patient data from the CDC and the Cleveland Clinic Registry database. Previous epidemiologic studies have identified age as an important risk factor for severe COVID‐19 (O'Driscoll et al., 2021; Williamson et al., 2020; Wingert et al., 2021), and our large COVID‐19 registry data further confirmed the elevated likelihood of severe COVID‐19 in aged individuals even after adjusting for sex, race, smoking, and multiple disease comorbidities (Figure 1c). Using the available laboratory testing data at the Cleveland Clinic COVID‐19 registry database, we found that aged severe COVID‐19 patients showed elevated levels of D‐dimer, CRP (Figure 1d), and NLR (Figure 1e). D‐dimer, CRP, and NLR are inflammatory markers associated with severity and death in COVID‐19 (Cai et al., 2021; Xu et al., 2020). These new findings that the increased incidence and severity of COVID‐19 are significantly associated with elevated inflammation motivate us to further identify age‐related immune cell subpopulations using large‐scale, single‐cell transcriptomics data from the patients with varying degrees of biology and clinical characteristics of COVID‐19.

Currently, Delta is the dominant variant of SARS‐CoV‐2 in the United States. Thus, we further inspected the odds ratio of hospitalization in COVID‐19 patients who carried different variants from children to aged populations using the CDC dataset since 1 January 2021 (Figure S1c). We found that younger COVID‐19 patients carried Delta variant were significantly associated with the increased likelihood of hospitalization. However, we observed no significant difference on hospitalization rate of COVID‐19 patients in both children and aged groups during the Delta variant prevalence period. There are several possible explanations. Fully vaccinated rate of ≥65 years aged individuals (85.8%) is 15% higher than that of younger individuals (70.3%) since 11 November 2021 (https://covid.cdc.gov/covid‐data‐tracker/#vaccinations_vacc‐total‐admin‐rate‐total). Children under 10 years have much lower incidence of COVID‐19 (Irfan et al., 2021). Further investigation of unique immune mechanisms of children under the resilience of COVID‐19 may provide novel age‐specific mechanisms in the future.

**FIGURE 6:**
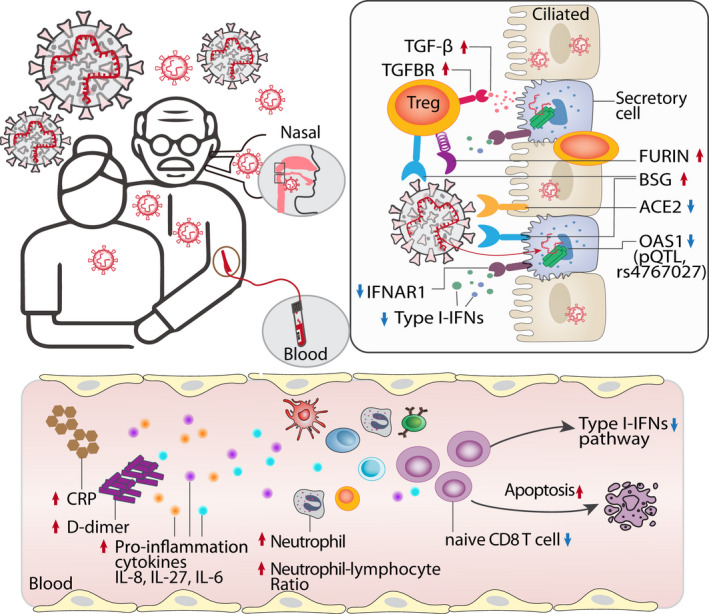
Proposed mechanistic models for age‐biased COVID‐19 severity in aged individuals. Several age‐related pathophysiologic immune responses are associated with disease susceptibility and severity in COVID‐19: a) decreased lymphocyte count and elevated inflammatory markers (C‐reactive protein [CRP], D‐dimer, and neutrophil–lymphocyte ratio); b) elevated pro‐inflammation cytokines IL‐8, IL‐27, and IL‐6 in aged COVID‐19 patients; c) reduced abundance of naïve CD8 T cells with decreased expression of antiviral defense genes (i.e., *IFITM3* and *TRIM22*) in aged individuals with severe COVID‐19; d) type I interferon deficiency is associated with SARS‐CoV‐2 viral load in aged individuals; e) elevated expression of SARS‐CoV‐2 entry factors (*BSG* and *FURIN*) and reduced expression of antiviral defense genes (*IFNAR1*, *OAS1*, *IFIT1*) in the secretory cells of critical COVID‐19 in aged individuals; f) strong TGF‐beta‐mediated immune–epithelial cell interactions (i.e., secretory—nrMa) in aged individuals with critical COVID‐19

Via deep immune cell profiling data analysis, we identified distinct immune responses in younger and aged COVID‐19 patients (Figure 6). For example, both younger and aged COVID‐19 patients showed increased ncMono cells and elevated IL‐6 (Figure 2 and Figure 6), while only aged COVID‐19 patients displayed elevated plasma IL‐8 and IL‐27 (Figure 2h). IL‐6 is a potential therapeutic target since it is a critical mediator of cytokine storm in COVID‐19 (Zhang, Wu, et al., 2020). However, a recent phase III clinical trial (NCT04320615) showed no reduced mortality in severe COVID‐19 patients treated with the anti‐IL‐6R monoclonal antibody tocilizumab (Rosas et al., 2021). Younger COVID‐19 patients in the ICU also showed significantly higher IL‐10 (Figure 2h). Our observations suggest that targeting IL‐10 might reduce mortality in younger patients with severe COVID‐19. Furthermore, an anti‐IL‐8 drug (BMS‐986253) is under testing for COVID‐19 patients in a Phase 2 clinical trial (ClinicalTrials.gov Identifier: NCT04347226). Therefore, our findings suggested that age is an important biological variable in evaluation of clinical benefits of anti‐IL‐8 intervention trials.

We also found reduced lymphocytes in hospitalized aged COVID‐19 patients (Figure 1d). In particular, the abundance of naïve CD8 T cells was decreased in aged patients with severe COVID‐19 (Figure 2d). Reduction of naïve CD8 T cell is a hallmark of immunosenescence in older individuals (Goronzy et al., 2015), and through scRNA‐seq data analysis, we observed significant enrichment of up‐regulated apoptosis genes in CD8 naïve T cells from aged COVID‐19 patients. Mechanistically, the apoptosis driver gene *CTSD* (Cocchiaro et al., 2016) is significantly elevated in naïve CD8 T cells from aged severe/critical COVID‐19 patients compared with younger patients (*q* < 2.0 × 10^−16^). Thus, modulation of CD8 naïve T‐cell dysfunction, especially targeting apoptosis pathway (Chu et al., 2021), may provide a new treatment strategy for severe COVID‐19 in aged patients.

IFN‐mediated immunity provides initial rapid protection against viral infection (McNab et al., 2015), and about 3.5% of patients with life‐threatening COVID‐19 show genetic aberrations in the type I IFN pathway (Zhang, Bastard, et al., 2020). A recent genetic study in European ancestry revealed that the cis‐protein quantitative trait loci (pQTL, rs4767027) in *OAS1* (an IFN‐stimulated gene) were significantly associated with decreased likelihood of COVID‐19 susceptibility and severity (Zhou et al., 2021). Herein, we found that aged individuals with severe COVID‐19 show reduced expression of type I IFN genes (Figures 3b,c, 4a, and 5b). Notably, aged patients with high SARS‐CoV‐2 viral load show reduced expression of *OAS1* and *IFNA1*, *IFNA5*, *and IFNA7* (Figure 4a) compared with younger patients. On the contrary, aged patients with high SARS‐CoV‐2 viral load have elevated expression of the pro‐inflammatory cytokine IL‐8 and decreased lymphocyte cell counts in plasma (Figure 4c), demonstrating dysregulation of cytokine responses that has been well described for COVID‐19 (Acharya et al., 2020). Of note, the dysregulated cytokine response is likely the effect of a variety of immunomodulatory strategies employed by SARS‐CoV‐2 that are used to manipulate specific signaling pathways that lead to cytokine induction such as the RIG‐I‐like receptor pathway. Now, there are more than 40 ongoing clinical trials (https://clinicaltrials.gov/) to test interferon‐related therapies for potential treatment of COVID‐19. Our findings suggested that interferon‐related therapies may provide more clinical benefits for older individuals with COVID‐19.

Although aged adults show increased susceptibility to SARS‐CoV‐2 infection compared to children (Davies et al., 2020), we did not find differences in SARS‐CoV‐2 viral load in the upper airways between younger and aged patients (Figure S8). Using large‐scale scRNA‐seq data analysis, we did find, however, that the SARS‐CoV‐2 entry genes (*ACE2*, *BSG*, *TMPRSS2*, *FURIN*, and *NPR1*) showed cell type‐specific expression profiles in both aged and younger individuals. In aged patients with critical COVID‐19, the expression of BSG was increased in secretory, nrMa and CD8 T cells, and elevated expression of FURIN was found in Treg and CD8 T cells. Thus, cell type‐specific host factor expression may play an important role in age‐mediated disease susceptibility and severity in COVID‐19.

We also identified age‐specific increases in immune–epithelial cell interactions. For example, we found strong TGF‐β‐mediated immune–epithelial cell interactions in aged severe COVID‐19 patients (Figure 5b and Figure S7). TGF‐β plays a crucial role in pulmonary fibrosis (Khalil et al., 1991; Lee et al., 2001), which is a common complication in severe COVID‐19 patients (Leeming et al., 2021). The nucleocapsid protein of SARS‐CoV‐1 also upregulates TGF‐β expression (Zhao et al., 2008). Thus, TGF‐β‐targeted therapies may provide better clinical benefits for aged patients with COVID‐19. We additionally identified receptor‐interacting serine/threonine kinase 1 (RIPK1)‐mediated immune–epithelial cell interactions (secretory/ciliated–CD8 T‐cell pairs) in younger patients with critical COVID‐19. RIPK1 is a key mediator of inflammation (Mifflin et al., 2020), and a RIPK1 inhibitor (SAR443122) has been tested in a phase I clinical trial (ClinicalTrials.gov Identifier: NCT04469621) to treat tissue damage resulting from inflammation in severe COVID‐19 patients. Altogether, RIPK1 inhibitors (Riebeling et al., 2021) may offer a potential treatment for young COVID‐19 patients, such as COVID‐19‐related multisystem inflammatory syndrome in children (MIS‐C) (Rowley, 2020).

Lastly, we acknowledge the potential limitations of our study. Although we inspected omics data from multiple tissues, including PBMCs, plasma, and nasal tissues, additional analysis of other COVID‐19 and aging relevant tissues, such as lung and brain, should be investigated in the future. In addition, our COVID‐19 database and omics data were generated from acute COVID‐19 patients, and identification of the underlying genetic and molecular basis of aging differences for long‐haul COVID‐19 patients will be an important area of future investigation (Sudre et al., 2021). As the inconsistent correlation between RNA expression and protein expression (Buccitelli & Selbach, 2020), further investigation of differential protein expression of ACE2, BSG receptors, and the TGF‐β using proteomics data is highly warranted in the future studies. Finally, investigation of COVID‐19 vaccine responses between aged and young patients is also warranted in the future.

## 4 EXPERIMENTAL PROCEDURES

“Younger” was defined as 18 to 55 years of age, and “aged” was defined as ≥65 years old.

### 4.1 U.S. CDC COVID‐19 epidemiological data

Publically accessible COVID‐19 death counts in 54 states and territories in United States were downloaded from the CDC Web site (https://data.cdc.gov/NCHS/Provisional‐COVID‐19‐Death‐Counts‐by‐Sex‐Age‐and‐S/9bhg‐hcku/data) on 23 December 2020 (Table S1). Publically accessible statistics of influenza mortality across 10 flu seasons (November 2010–2020) in United States was downloaded from CDC Web site (https://catalog.data.gov/dataset/deaths‐from‐pneumonia‐and‐influenza‐pi‐and‐all‐deaths‐by‐state‐and‐region‐national‐center‐) on 20 June 2020. Both COVID‐19 and influenza datasets include three age‐stratified groups: 0–17 years, 18–64 years, 65 years, and older. These datasets were used for epidemiological prevalence analysis of COVID‐19 and influenza.

We collated U.S. COVID‐19 Case Surveillance Public Use Data from the CDC website (https://healthdata.gov/dataset/covid‐19‐case‐surveillance‐public‐use‐data) from December 2019 to 28 December 2020. This dataset includes age‐stratified COVID‐19 case counts in hospitalization, ICU admission, death, sex, and race. We extracted two age subgroups from all laboratory‐confirmed cases using the following criteria: i) the age range of younger group from 20 to 49 years and the age range of older group over than 60 years (Table S2); ii) deletion of all cases in which sex and race information was missing. In total, the younger subgroup includes 2,369,919 cases, with 94,161 in hospitalization, 9138 in ICU admission, and 6469 death cases. The older subgroup has 1,048,011 cases in total, with 243,109 in hospitalization, 29,671 in ICU admission, and 124,566 death cases. This dataset was used to determine OR analysis.

### 4.2 COVID‐19 registry database

We used institutional review board–approved COVID‐19 registry data, including 45,077 individuals (12,651 aged patients and 32,426 younger patients; Table S3) tested during March to December, 2020 from the Cleveland Clinic Health System in Ohio and Florida. All tested samples were pooled nasopharyngeal and oropharyngeal swab specimens. Infection with SARS‐CoV‐2 was confirmed by RT‐PCR in the Cleveland Clinic Robert J. Tomsich Pathology and Laboratory Medicine Institute. In total, 12,304 patients (aged *n* = 3559, younger *n* = 8745) tested COVID‐19 positive by the end of December 2020. All SARS‐CoV‐2 testing was authorized by the Food and Drug Administration under an Emergency Use Authorization, in accord with the guidelines established by the Centers for Disease Control and Prevention.

The data in COVID‐19 registry include COVID‐19 test results, baseline demographic information, and all recorded disease conditions (Table S3). We conducted a series of retrospective studies to test the association of aging with COVID‐19 outcomes, including hospitalization, ICU admission, mechanical ventilation, and death. Data were extracted from electronic health records (EPIC Systems), and patient data were managed using REDCap electronic data capture tools. To ensure data quality, a study team trained on uniform sources for the study variables manually checked all datasets. Statistical analysis for smoking, hypertension, diabetes, coronary artery disease asthma, and emphysema and COPD was calculated after missing value deletion.

### 4.3 Clinical outcome analysis

The OR was used to measure the association between COVID‐19 outcomes and aging based on logistic regression. An OR >1 indicates that aged patients are associated with a higher likelihood of the outcome. To reduce the bias from confounding factors, we employed OR analysis in two datasets. For U.S. CDC datasets, the OR model was adjusted by sex and race, due to limited information of other confounding factors. However, in the COVID‐19 registry, we adjusted for sex, race, smoking, hypertension, diabetes, coronary artery disease, asthma, emphysema, and COPD. The Kaplan–Meier method was used to estimate the cumulative hazard of hospitalization of COVID‐19 patients across age groups. For hospitalization outcome, the time was calculated from the start date of COVID‐19 symptoms to hospital admission date. Log‐rank test was used for comparison across different age groups with Benjamini and Hochberg adjustment (Benjamini & Hochberg, 1995). Cumulative hazard analysis was performed using the Survival and Survminer packages in R 3.6.0 (https://www.r‐project.org).

### 4.4 Public available COVID‐19 multi‐omics datasets used in this study

Detailed information of the list datasets shown in Table S1.

### 4.5 Two single‐cell sequencing datasets

In this study, we used two COVID‐19 single‐cell datasets (Table S1). 1) The CD8^+^ T‐cell dataset (Stephenson et al., 2021) is a sub‐dataset from original PBMC single‐cell data. We re‐analyzed 59,815 single‐cell transcriptomes of CD8 T cells, which revealed 5 distinct CD8 sub‐clusters (Figure 3a), including CD8 naïve (CCR7^+^, LEF1^+^), Tcm (GZMK^+^, LTB^+^, CCR7^−^), Tem (GZMK^+^, CCR7^−^), CD8 proliferation (MKI67), and CD8 T terminal effector cell (also named CD8 TEMRA (Thome et al., 2014), HLA‐DRB1+, GZMB+, GNLY+, LAG3+). Based on our aging criteria, the critical/severe COVID‐19 patients were grouped to aged (*n* = 12) and younger patients (*n* = 13). 2) A single‐cell dataset from nasal tissues (Chua et al., 2020) (European Genome‐phenome Archive repository: EGAS00001004481) was from COVID‐19‐positive patients (11 critically ill patients and 8 moderately ill patients). Based on our aging criteria, we extracted a subpopulation from the original cohort. The final COVID‐19 cohort used in this study included 8 critically ill patients (5 younger and 3 older patients) and 7 moderately ill patients (4 younger and 3 older patients). As the original dataset supplied cell type information, additional analysis was based on cell type annotation. The dataset contained 115,895 cells across 15 cell types (B cell, Basal, Ciliated, Ciliated‐diff, CD8 T cell, moDC, Neu, NKT, NKT‐p, nrMa, rMa, Secretory, Secretory‐diff, Squamous, and Treg).

### 4.6 Bulk RNA‐sequencing dataset in nasal tissue (Lieberman et al., 2020)

The dataset was publically available from NCBI GEO database (GSE152075). Based on original meta‐information, we extracted COVID‐19‐positive sample data with high or low viral load, deleting samples in which sex and age information were missing. 147 bulk RNA‐seq samples were used in this study, including 61 aged patients (high viral load *n* = 27, low viral load n =34) and 86 younger patients (high viral load *n* = 46, low viral load *n* = 40).

### 4.7 SARS‐CoV‐2 viral load dataset (Fajnzylber et al., 2020)

We quantified SARS‐CoV‐2 RNA load from 5 specimen types, including upper airway specimens (oropharyngeal swab [detectable percentage was 67%], nasopharyngeal [detectable percentage was 50%], sputum [detectable percentage was 85%]), plasma [detectable percentage was 27%], and urine [detectable percentage was 10%]). We selected hospitalized patients with at least one COVID‐19‐positive test among upper airway or plasma specimens. Finally, 72 patients were used for correlation analysis between age and viral loading. 43 patients (older patients *n* = 18, younger patients *n* = 25) with SARS‐CoV‐2 RNA detectable testing in upper airway were used to analyze the change of clinical inflammatory variables in both aged and younger groups. In our study, 54 patients tested positive for plasma SARS‐CoV‐2 RNA, including 21 patients with SARS‐CoV‐2 RNA (aged patients *n* = 13). There were 35 SARS‐CoV‐2 RNA undetectable patients (aged patients *n* = 7).

### 4.8 Circulating cell flow cytometry datasets (Takahashi et al., 2020)

This dataset included 12 major immune cell types as a percentage of PBMC and 32 T‐cell subtypes as a percentage of CD3‐positive cells through flow cytometry (Table S5). It also detected the plasma concentration of 71 cytokines through cytokine array. Based on our age criteria, the dataset included 81 hospitalized patients, 40 with longitudinal data. When the second follow‐up time of a patient was greater than 7 days, it was recorded as two samples. Hence, 114 samples were analyzed, which included 94 older samples (non‐ICU *n* = 66, ICU = 26) and 50 younger samples (non‐ICU *n* = 37, ICU = 13).

### 4.9 Single‐cell sequencing data analyses

All single‐cell data analyses and visualizations were performed with the R package Seurat v3.1.4 40. The data quality filtering was strictly followed by the original literature (Chua et al., 2020; Ren et al., 2021). “NormalizeData” was used to normalize the data. “FindIntegrationAnchors” and “IntegrateData” functions were used to integrate cells from different samples. Principal component analysis (PCA) and Uniform Manifold Approximation and Projection (UMAP) with 15 principal components were used. A resolution of 0.5 was used in “FindClusters()” step. “FindAllMarkers” function with the MAST test was employed as the finding maker method for each cell type.

### 4.10 Cell–cell interaction analysis

Cell–cell interaction analysis was based on normalized expression data of known ligand–receptor pairs in 15 cell types of nasal single‐cell sample. The analysis was performed by CellPhoneDB (Efremova et al., 2020) v2.1.4 (https://github.com/Teichlab/cellphonedb) based on the python 3.7 platform. Statistical analysis mode was used to identify significant ligand–receptor pairs in each cell number. A permutation test (1000 randomizations) with BH multiple testing correction was used to evaluate the significance.

### 4.11 Bulk RNA‐sequencing data analysis

All bulk RNA‐sequencing data analysis started from raw counts value. R package edgeR (Robinson et al., 2010) v3.12 was used to analyze differentially expressed genes in older vs. younger groups. Correction for sex and batch effects was added into the formula of design model. Statistical significance *p*‐values were adjusted by BH (*q* value) method (Benjamini & Hochberg, 1995). Differentially expressed genes were identified as adjusted *p*‐value (*q*) <0.05 and log‐fold change >0.5.

### 4.12 Immune gene set enrichment analysis

To evaluate the immune pathway profiles in young and aged COVID‐19 patients, GSEA was conducted as previously described (Subramanian et al., 2005). Immune gene profiles were retrieved from the KEGG database (Kanehisa et al., 2017). We selected 22 immune‐related pathways and 1241 genes from KEGG belonging to the immune system subtype. For each cell type, we performed a GSEA on the list of differential expressed genes (DEGs) ranked by the log_2_FC. The normalized enrichment score (NES, Equation 1) was calculated for 22 immune pathways in young and aged specific gene sets (Figure 4b),

(1) $$\text{NES =}\;\frac{\text{ES}}{\bar{{\text{ES}}_{\text{permutation}}}}$$

in which ES (Subramanian et al., 2005) denotes enrichment score. Normalization of the enrichment score reduced the effect of the differences in gene set size and in correlations between gene sets and the expression dataset. NES score >0 and *q* < 0.05 indicate that up‐regulated DEGs in aged vs. young are significantly enriched in immune pathways, while NES score <0 and *q* < 0.05 indicate down‐regulated DEGs in aged vs. young are significantly enriched in immune pathways. Permutation test (1000 times) was performed to evaluate the significance. All analyses were performed with the prerank function in GSEApy package (https://gseapy.readthedocs.io/en/master/index.html) on Python 3.7 platform.

### 4.13 Statistical analysis

Statistical tests for assessing categorical data through chi‐square test and the two‐tailed Mann–Whitney *U* test were used to compare the difference in continuous variable by aged vs. younger. Spearman's ρ was assessed for correlation between two variables. Statistical significance level was set at *q* < 0.05 and corrected by Benjamini–Hochberg (false discovery rate) method. All statistical analysis was performed by SciPy Statistics (https://docs.scipy.org/doc/scipy/reference/stats.html#module‐scipy.stats).

## CONFLICT OF INTEREST

The authors declare that they have no competing interests.

## AUTHOR CONTRIBUTIONS

F.C. conceived the study. Y.H. performed all data processing and all experiments. Y.Z., M.U.G., Y.L., L.J., T.A.C., H.Y., C.E., and A.A.P. discussed and interpreted results. Y.H., A.A.P., and F.C. wrote the manuscript and all authors critically revised the manuscript and gave final approval.

## Supporting information

Fig S1‐S8

Table S1‐S7

## Data Availability

The clinical and transcriptomic datasets used in this study are publicly available; for details, see Table S1. The code for single‐cell analysis can be found in https://github.com/ChengF‐Lab/COVID‐19_Map.
