# Supplementary material for: Aging‐related cell type‐specific pathophysiologic immune responses that exacerbate disease severity in aged COVID‐19 patients
Source: Aging Cell. 2022 Jan 12;21(2):e13544. doi: 10.1111/acel.13544 (PMC8844132; doi:10.1111/acel.13544)
Supplement: Supplementary file 1 — Fig S1‐S8 [file ACEL-21-e13544-s002.pdf]

Figure S1

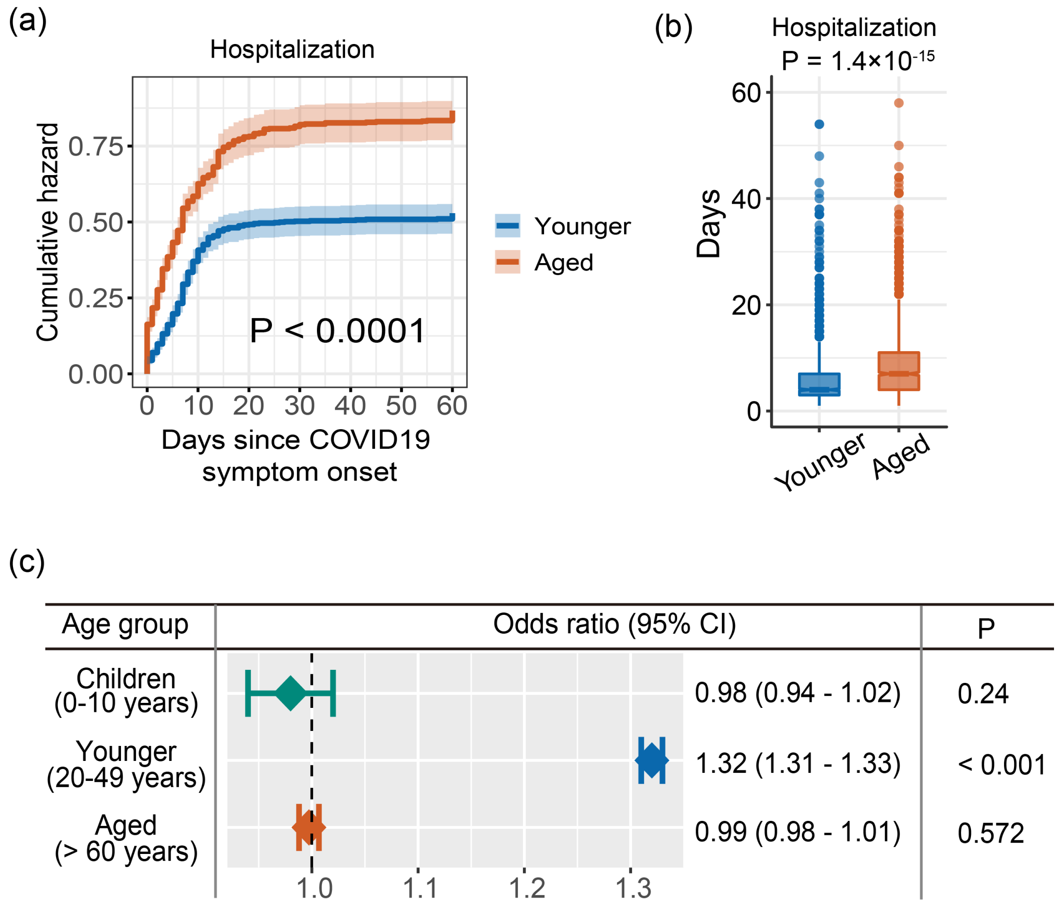

FIGURE S1. Aged COVID-19 patients with elevated hazard of Hospitalization. (a) Cumulative hazard of COVID-19 hospitalization. The log-rank test with the Benjamini & Hochberg (BH) adjustment are used to compare the statistical significance of cumulative hazard of hospitalization. The shadow represents 95% confidence interval. (b) Boxplots of the straying duration in hospital between aged and younger patients. Statistical p-value was computed by Mann–Whitney U test. (c) Odds ratio (OR) analysis of COVID-19 hospitalization patients during Delta variant prevalence period. U.S. CDC dataset from 2021 Jan 1st to 2021 Oct 14 were used to analyze OR. Delta variant causes more than 90% of new COVID-19 cases in US from 2021 July. Thus, we split CDC dataset to two subsets from 2021 July 1st. The cases diagnosed before July 1st were defined as Delta variant recessive period; and the cases diagnosed after July 1st were defined as Delta variant dominant period. OR > 1 indicates that COVID-19 patients in Delta variant dominant period have an increased likelihood of hospitalization. The colors denote OR models in different age stratification.

Figure S2

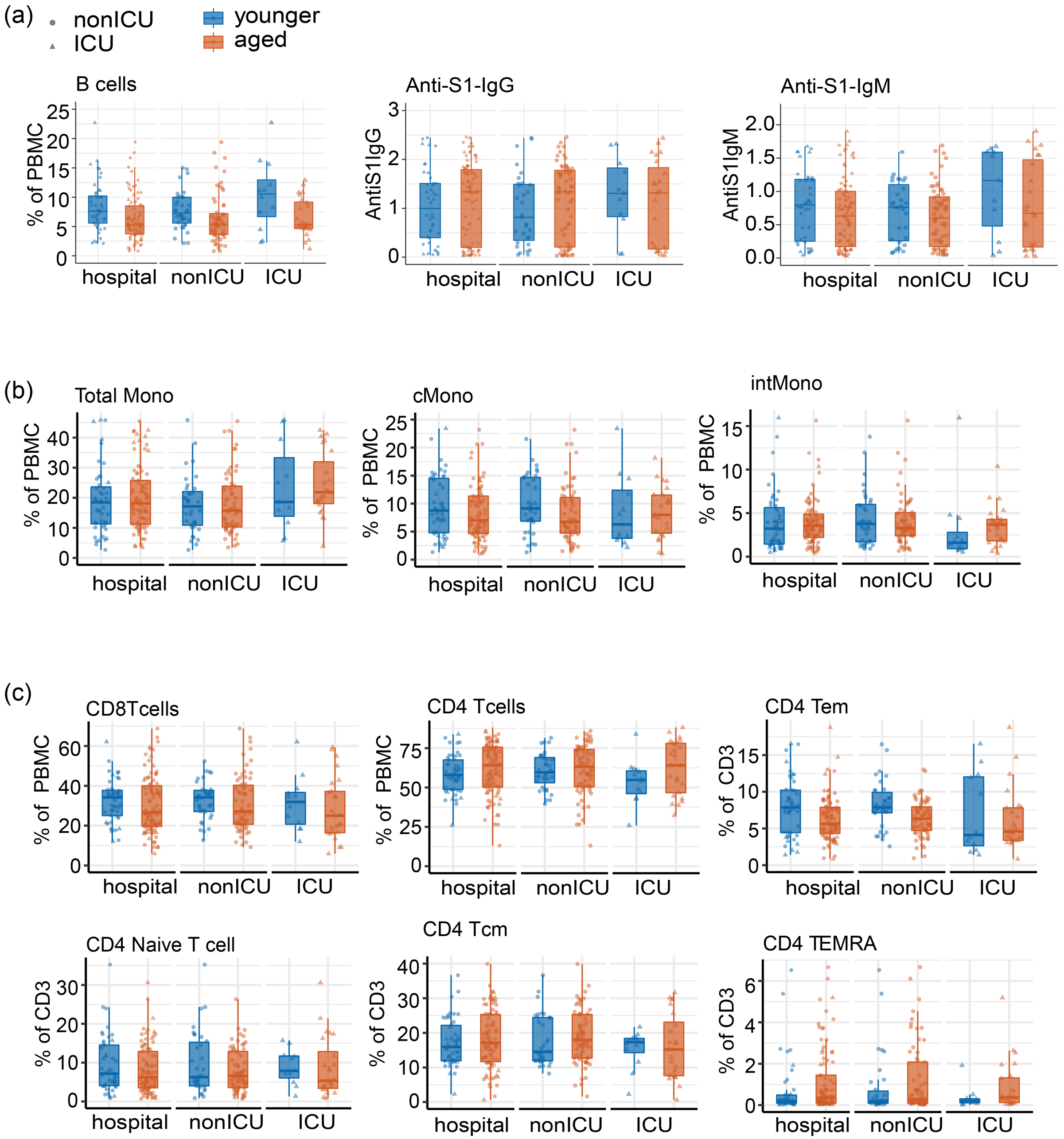

FIGURE S2. Comparison of the abundance of B cells in PBMCs and cytokines in plasma between aged and younger COVID-19 patients. (a) and (b)The abundance of B cells (a) and monocyte cells (b) in PBMC. (c) The abundance of CD8 and CD4 subtype in CD3 positive. Statistical p-value was computed by Mann–Whitney *U* test.

Figure S3

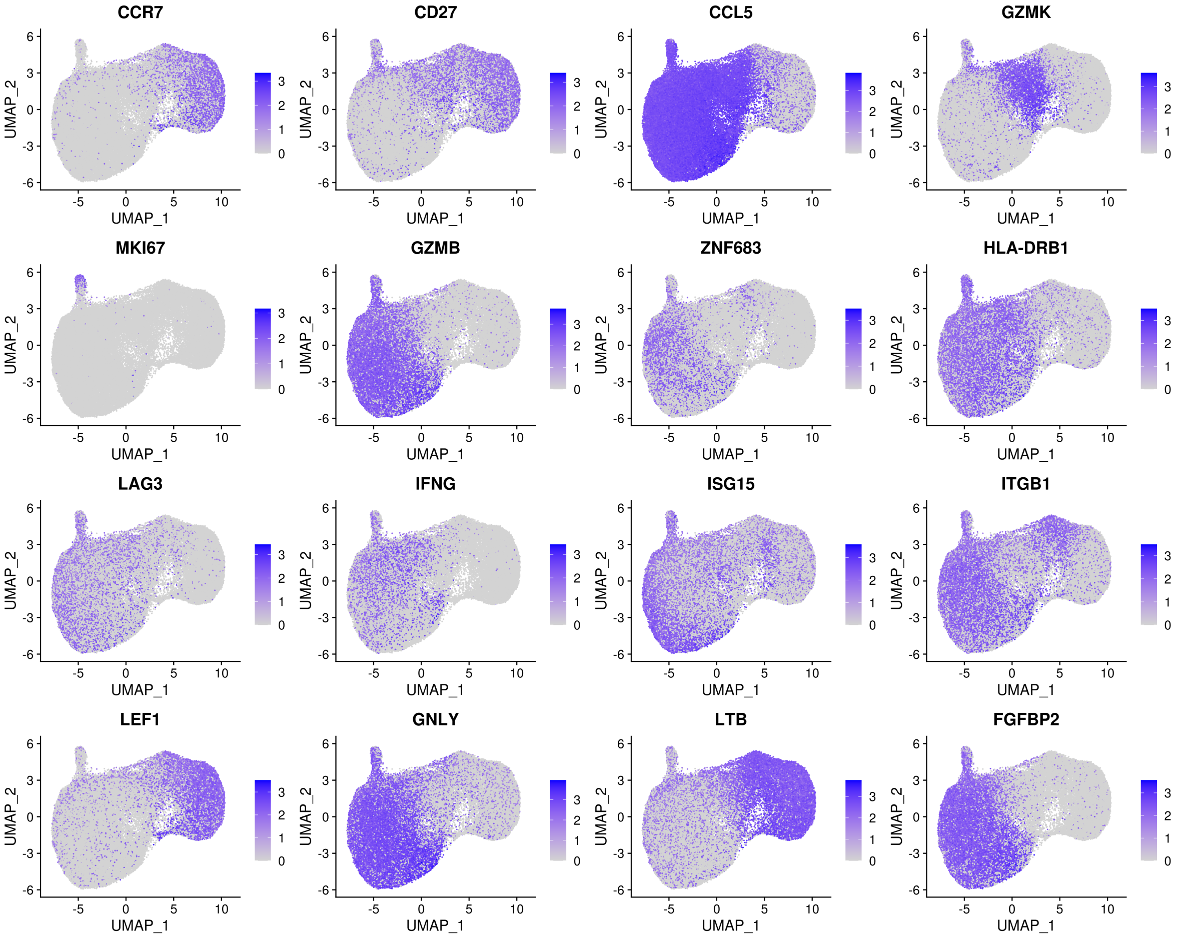

FIGURE S3. The expression of marker genes shown on the UMAP plot. The expression levels are blue color coded.

Figure S4

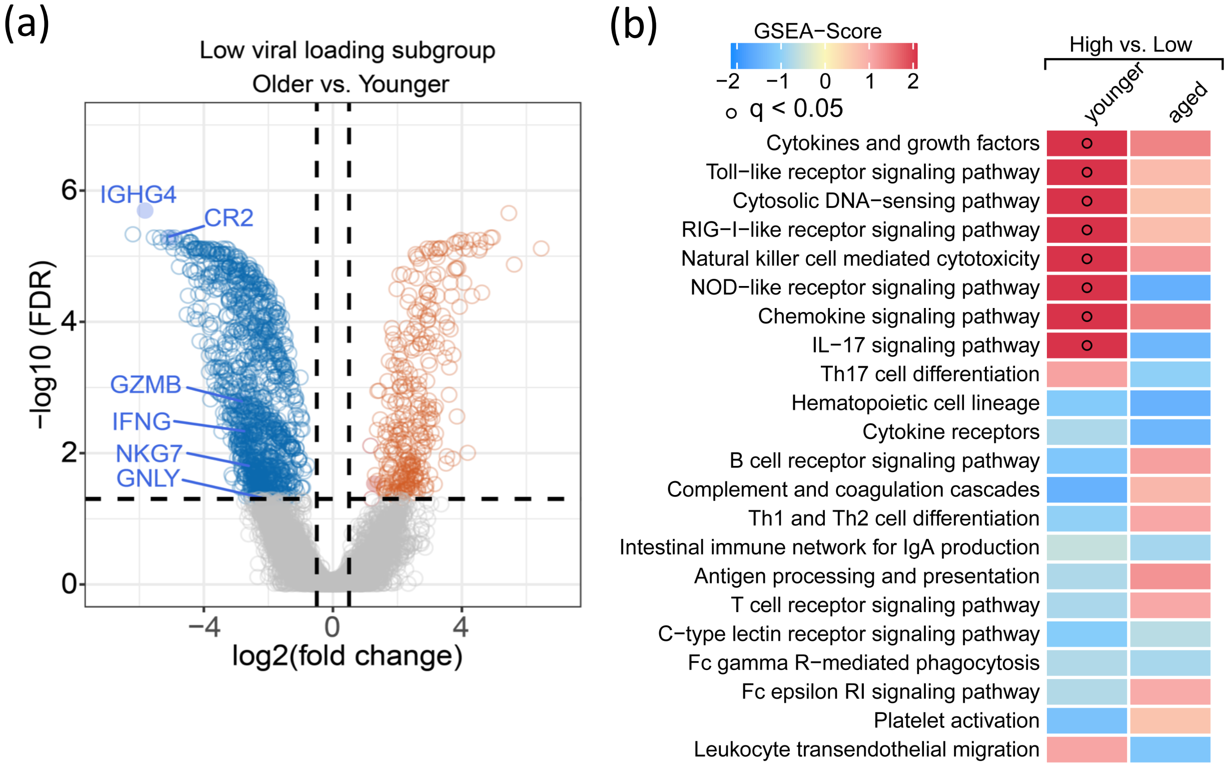

FIGURE S4. Analysis of relationship between age and SARS-CoV-2 viral load in nasal tissues. (a) Volcano plot show the differential genes of bulk RNA-sequencing data in aged versus younger in low viral load nasal tissues. (b) Gene-set enrichment analysis (GSEA) across 22 immune pathways for differential genes of high vs low viral load in aged or younger subgroups. The gradient color bar shows the NES score (see Method). NES score > 0 and q < 0.05 indicates that up-regulated DEGs in high vs. low are significantly enriched in immune pathways, while NES score < 0 and q < 0.05 indicates that down-regulated DEGs in high vs. low are significantly enriched in immune pathways. Black circle denotes q < 0.05.

Figure S5

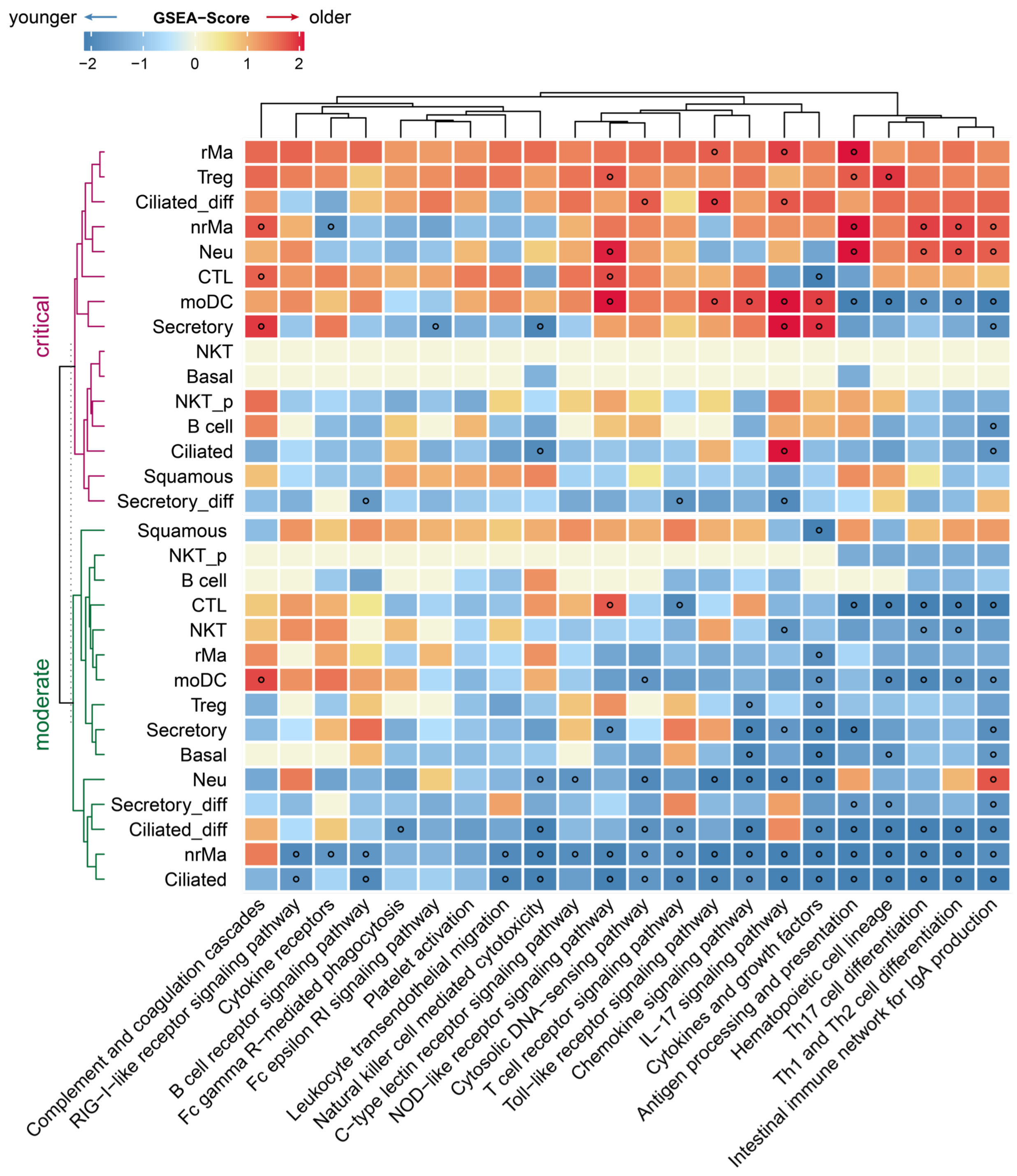

FIGURE S5. Gene-set enrichment analysis (GSEA) of 22 immune pathways across 15 cell types of nasal tissues. The gradient color bar shows the NES score (see Method). NES score > 0 indicates the immune pathway significantly enriched in upper-regulated genes. NES score < 0 indicates the immune pathway significantly enriched in down-regulated genes. Black dots denote the FDR < 0.05.

Figure S6

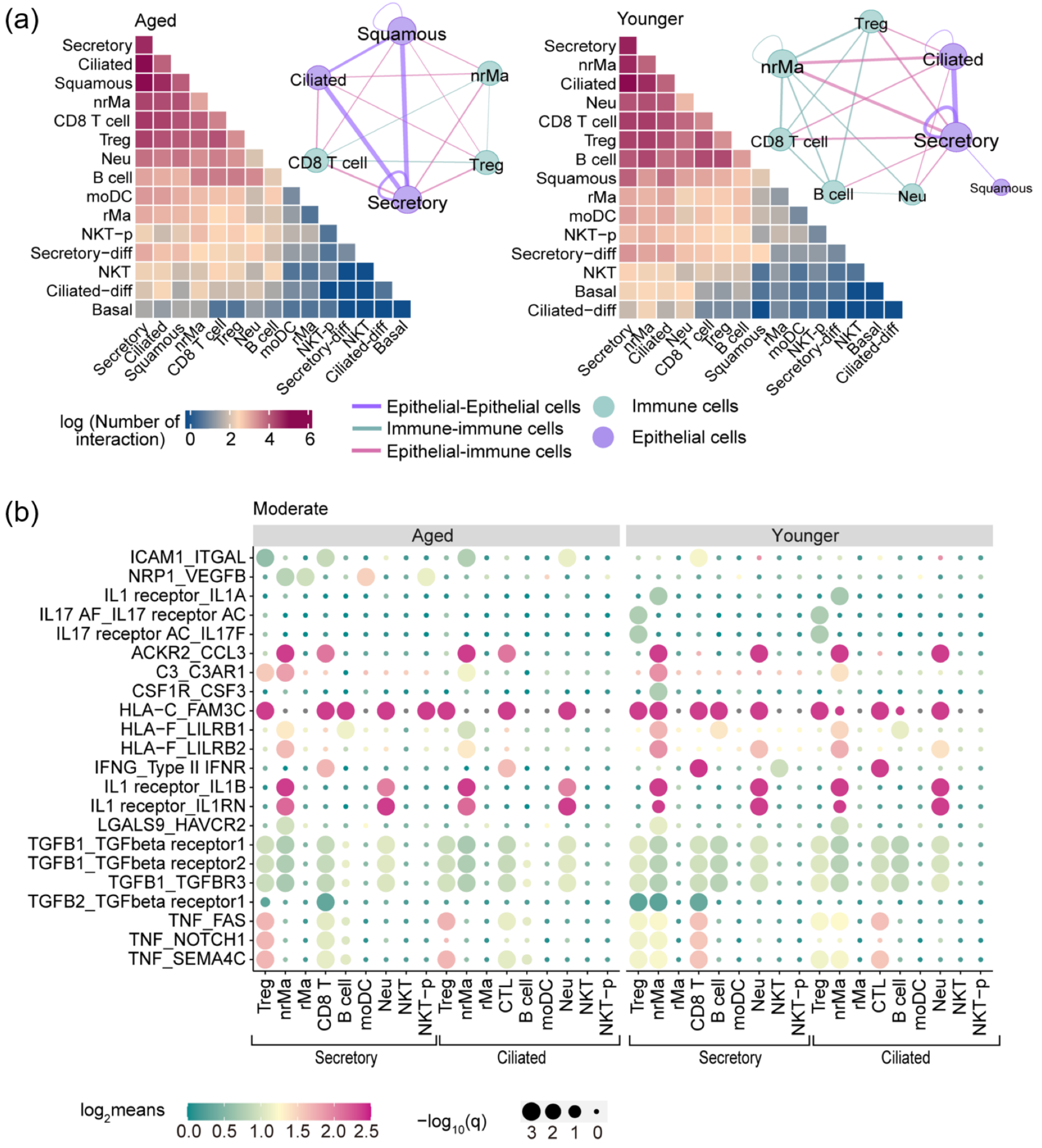

FIGURE S6. Distinct epithelial-immune cell interaction profile in aged and younger patients with moderate COVID-19. (a) Heatmap show the total log-scaled interaction number between epithelial-immune cells in moderate COVID-19 disease. Aged group, n= 3 patients, younger group, n = 4 patients. The cell-cell interaction network depicted all cell pairs which the number of cell-cell interaction > 50. Edge size denotes the number of interactions between two cell types. Different colors indicate the immune or epithelial cell types. (b) Dot plot showing the significant ligand-receptor interactions between epithelial-immune cell interaction in moderate COVID-19 disease. The circle size indicated -log10-scaled FDR by permutation test with BH multiple testing correction. Gradient color bar shows the log2-scaled means of average expression of interacted cell pair.

Figure S7

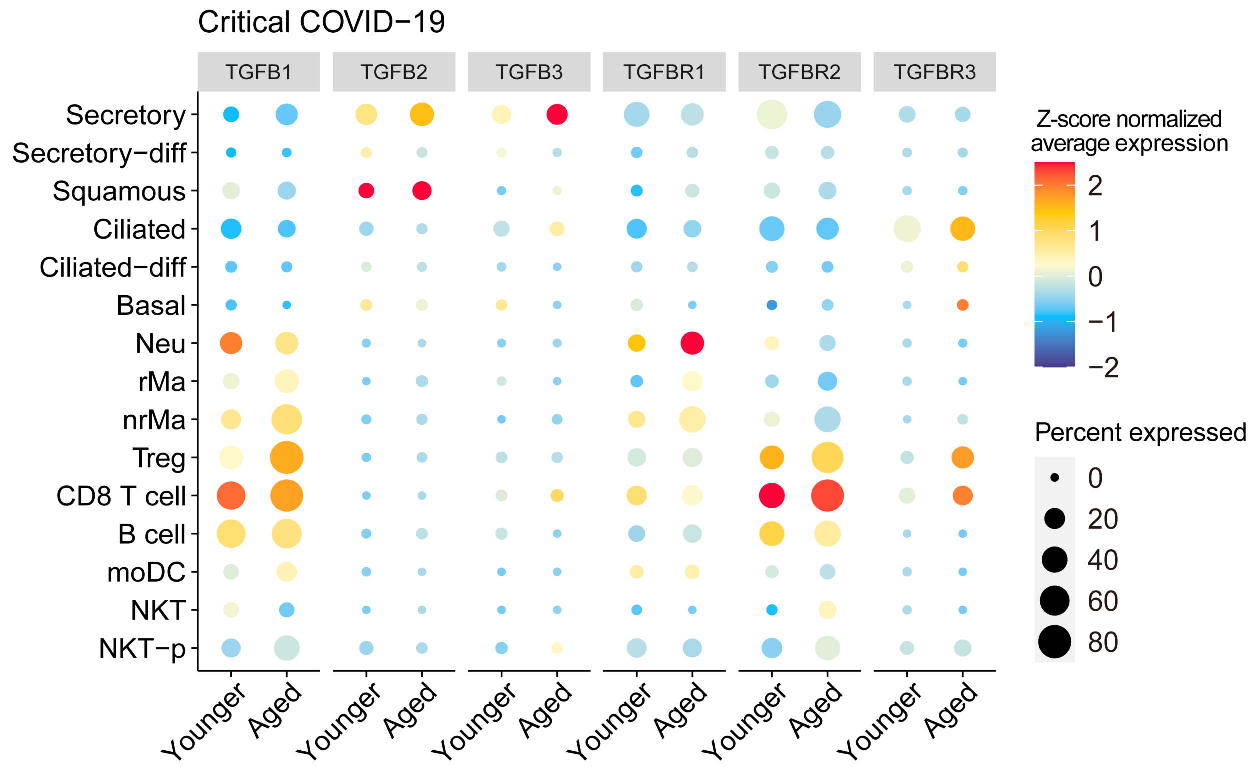

FIGURE S7. TGF-beta gene expression profile across 15 cell types of nasal tissue between aged and younger patients. The size of dot denotes the percentage of the positive cells expressing the tested genes. The gradient color bar represents the z-score scaled average expression of genes in each cell type.

Figure S8

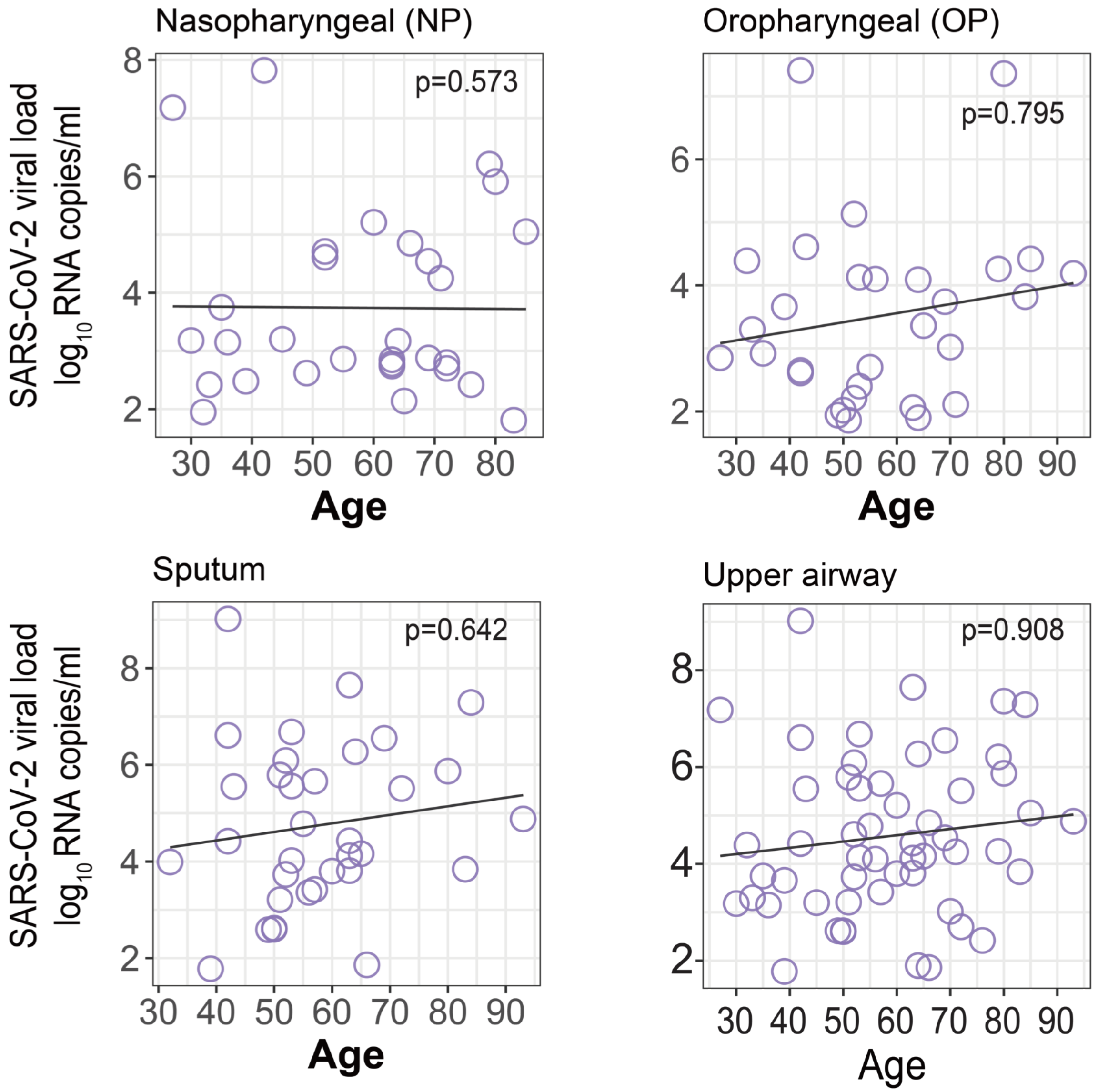

FIGURE S8. Correlation analysis between age and upper airway viral load. The upper airway data from three sample source, oropharyngeal swab, nasopharyngeal and sputum. The details of datasets are provided in the original literature (Fajnzylber et al., *Nature Communications*, 2020, 11:5493).
